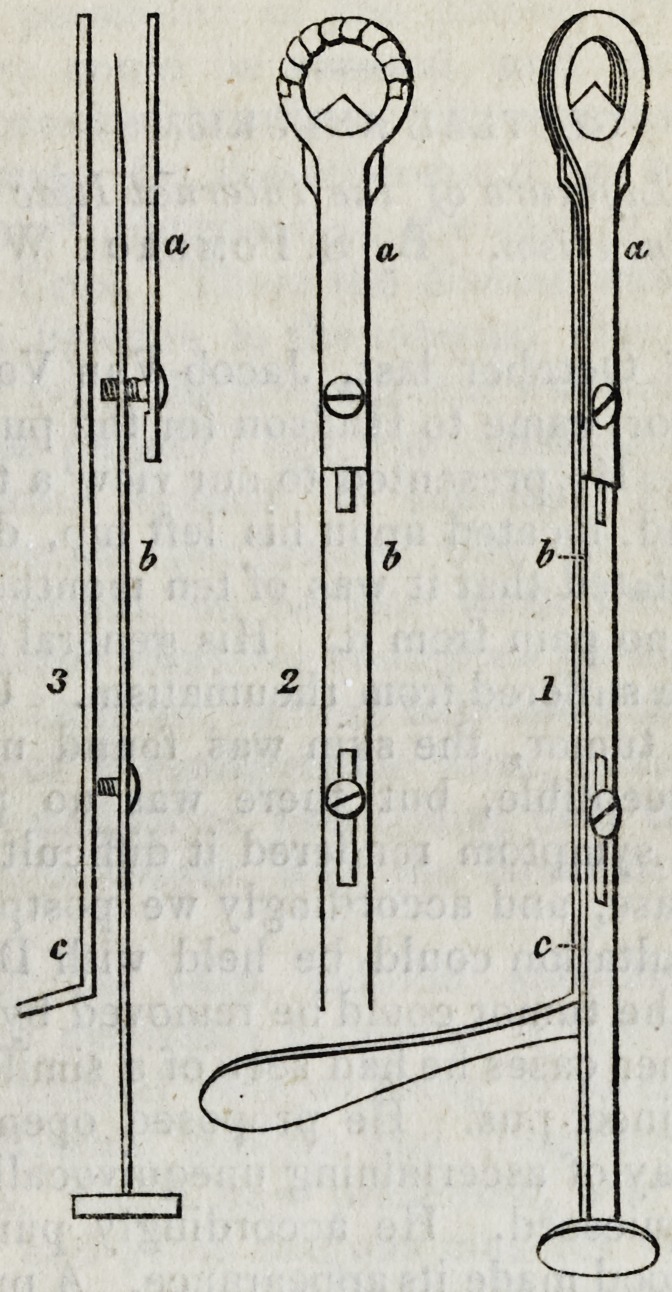# Case of Obstinate Cough, Occasioned by Elongation of the Uvula, in Which a Portion of That Organ Was Cut off

**Published:** 1828-07

**Authors:** Philip Syng Physick

**Affiliations:** Professor of Anatomy in the University of Pennsylvania.


### OBSTINATE COUGH.

*Case of Obstinate Cough, occasioned by Elongation of the Uvula, in which a Portion of that Organ was cut off.* By Philip Syng Physick, m.d. Professor of Anatomy in the University of Pennsylvania.

In June last, a young lady afflicted with a very obstinate cough applied to Dr. Physick, and gave him the following history of her case, drawn up by her physician at New Orleans:

" The first circumstances which had any connexion with the singular affection of this young lady, were a complaint of constant headache, attended with a disposition to vomit without nausea occurring first, during convalescence from an attack of remitting fever, in the middle of May, 1826. The latter symptom soon became the most prominent, and increased to a constant effort to retch, in which nothing was thrown up from the stomach, and which was not relieved by free vomiting. At this time no complaint of pain was made any where but in the head.

" Considering the gastric irritation as sympathetic of an incipient cephalic affection, leeches were applied to the temples and behind the ears, and some doses of active cathartic medicines given. No advantage was derived. The retchings became nearly constant, and, from a noisy effort to vomit, it gradually changed to a convulsive cough, altogether involuntary and uncontrolable, and conveying an impression as if something obstructed and irritated the organs of respiration. This, as nearly as it can be described, has been the character of the cough ever since.

" The first paroxysm increased in violence for a number of days, and until the 8th of September, when, about mid-day, after vomiting, (which was at this time not unusual with her,) in which she threw off a quantity of white tough mucus, she fell into a state of extreme prostration. The cough ceased, and she appeared to be dying. From this she slowly revived through the evening, and on the next day there was a degree of reaction amounting to fever, which gradually subsided, and left her quite well.

" The mucous expectoration, likewise, though at the time regarded with some interest, has, in the latter attacks, been produced occasionally in vomiting, but never followed by the same alleviation. On the recovery from the first attack, she remained well for two weeks, when she was again seized with the same spasmodic cough, attended with pain in the breast, but not preceded, as before, with any irritation of the stomach. This, after continually increasing in violence for about eight days, again left her in nearly the same manner it had done in the first instance. After an interval of three weeks, she had another attack of the same duration, and of extreme severity. Since this there has been two more, but at longer intervals, and not altogether of the same severity.

"The dates of the different paroxysms are the early part of September, of October, of November, of January, and of May. During the long interval between January and May, a slight cough of the same peculiar character has seized her every morning on awaking, after which she remains entirely exempt for the remaining twenty-four hours. At first it lasted for a few seconds only, but its duration gradually increased to thirty or forty minutes. Since the last violent attack, it has been reduced to only a few moments' continuance."

After many remedies had been used in the above case, without affording any permanent benefit, the patient was sent to Philadelphia, and Dr. Physick consulted. The circumstances appeared to him to point out an elongation of the uvula as the cause of the disease. On examining the throat, he found such an elongation actually existed. This was explained to the patient and to her friends, and the excision of a part of the uvula was performed; immediately after which all the symptoms ceased entirely, and have not since returned in the slightest degree.

In the operation of cutting off the uvula, Dr. Physick has, until very lately, used scissors; but being unable to complete the operation by one application of that instrument, several have been necessary to effect the division of the part. To obviate this difficulty, he determined to try the old instrument, as modified and represented by Benjamin Bell, in his System of Surgery. He found, however, that, although he could divide with that instrument the greater part of the uvula, a portion of the membrane that covers the back part of it was not always divided, making the use of scissors necessary to cut it through. To remedy this inconvenience, he caused an instrument to be made as represented in the annexed figure, having two plates instead of one, between which the knife was passed, but still the same difficulty was experienced in cutting through the membrane on its posterior part. He then thought of wrapping a strip of waxed linen over the semi-circumference of the opening, to support the membrane until it should be divided by the knife. Thus constructed, the instrument answered his purpose completely, and cut through the whole substance of the part in an instant. Dr. Physick has since used an instrument of similar construction for the removal of scirrhous tonsils. He finds it easy to cut off the whole, or any portion that may be necessary, of the enlarged tonsil in this manner. The operation can be finished in a moment of time. The pain is very little, and the hemorrhage so moderate that it has not required any attention in four cases in which the Doctor has lately performed it.

This instrument is so accurately represented in the annexed engraving, that a very brief description of it is all that can be required. Three views are given: 1st, a perspective; 2d, a front; 3d, a side view, the parts separated to show them more distinctly. The whole instrument is made of steel, and consists of two plates, *a* and *c*, between which is the cutting blade *b*. The upper plate, *a*, is short, and is fastened to the lower, *c*, by a screw, which passes through a groove in the blade *b*. The lower plate, *c*, is longer than the upper, and is bent at one end so as to form a handle. Between these two plates is the blade, *b*, one termination of which is somewhat lance-shaped and sharp, the other has a button on it, upon which the thumb is pressed when it is wished to push forward the blade. The blade is made to move steadily by the screw which connects together the upper and lower plate, and also by a second screw which passes through a groove in the blade, and fastens in the lower plate.

In figure 2, the strip of waxed linen is represented wound round the semi-circumference of the opening.

The size of the perforated end of the two plates, and of course that of the knife, must be larger in the instrument for extirpation of the tonsils, than in that for truncation of the uvula.*

* American Journal of the Medical Sciences.

## Figures and Tables

**Figure f1:**